# The need for a functional pharmaceutical industry in Sierra Leone: lessons from the COVID-19 pandemic

**DOI:** 10.1186/s40545-022-00444-w

**Published:** 2022-07-27

**Authors:** Eugene Conteh, Melody Okereke, Foday Umaro Turay, Alhaji Sununu Bah, Abdulwasiu Muhsinah

**Affiliations:** 1 Pharmaceutical Society of Sierra Leone, Freetown, Sierra Leone; 2 Faculty of Pharmaceutical Sciences, University of Ilorin, Ilorin, Kwara State Nigeria; 3 Department of Pharmaceutical Chemistry, Faculty of Pharmaceutical Sciences, College of Medicine and Allied Health Sciences, University of Sierra Leone, Freetown, Sierra Leone; 4 Dora Akunyili College of Pharmacy, Igbinedion University Okada, Edo State, Nigeria

## Abstract

With an estimated population of 8.3 million, Sierra Leone has no existing pharmaceutical manufacturing company at present. The recent COVID-19 pandemic brought to the limelight the fragility and weakness of the Sierra Leonean pharmaceutical industry as it resulted in severe drug shortages and medicine insecurity in the country due to the restrictions imposed on importations, which validates the uncertainties if the country can sufficiently meet its local pharmaceutical needs and health demands. In this paper, we highlight the current situation of the Sierra Leonean pharmaceutical sector and provide recommendations for the country towards building a functional pharmaceutical industry based on lessons learnt from the COVID-19 pandemic.

## Background

The pharmaceutical industry manufactures medicines, medical devices, and diagnostic tools for treating or preventing diseases. The global market value of the pharmaceutical sector is about US $1.42 trillion, making it a significantly huge industry [1]. The world’s major and most prominent pharmaceutical companies such as Pfizer, Merck, Johnson & Johnson are domiciled in the United States, while Sanofi, in France, and Novartis and Roche, in Switzerland [1]. It can therefore be asserted that North America and Europe remain one of the largest pharmaceutical submarkets in the world, which invariably attracts significant attention to the performance of the pharmaceutical industry in other continents such as the African continent.

For decades, the African continent has been confronted with the problem of severe drug shortages. According to the World Health Organization (WHO), about half of the total population in the continent do not have regular access to even the most important medicines [2]. This further supports the evidence revealed that the lack of access to medicines kills millions of people annually in Africa, and that the burden is primarily on the poor, women, and children [3]. Global estimates reveal that Africa accounts for 17% of the world population, but due to factors tied to its insufficient pharmaceutical manufacturing capacity, it currently covers only 1% of the total vaccination needs [4]. There are more than 50 countries in Africa, and only eight countries, Algeria, Egypt, Ethiopia, Morocco, Nigeria, Senegal, South Africa, and Tunisia, operate the entire vaccine manufacturing industry [5]. Of these eight countries, only Senegal has been granted pre-qualification by the WHO to export vaccines [5]. The others, on the other hand, cannot export although some countries in the continent have a small number of local pharmaceutical companies that manufactures essential drugs and other health commodities for the local market.

In total, there are about 375 pharmaceutical companies in the continent, mainly in North Africa, and serving about 1.3 billion people [6]. However, Africa still relies heavily on imported medicines to meet the health needs of the region. In 2019, 70–90% of the medicines consumed in the estimated US $14 billion drug market in sub-Saharan Africa were imported [6]. In addition, Africa accounts for almost 25% of the global vaccine demand, but produces only 0.1% of the world's vaccines [6]. For context, in Africa, 99% of COVID-19 vaccine doses are imported and the majority of the 1% (12 million doses) produced domestically only undergo final filling, packaging, and finishing stages [4], without any actual vaccine manufacturing taking place.

While the spotlight has always been centered on the African continent as a whole, current developments in the pharmaceutical industry reinforce the need to focus on country-specific measures and roadmaps in a bid to provide further insights and understanding on how functional the pharmaceutical industry of every country in the continent is in meeting domestic health needs, targets and priorities. One of such countries worth exploring is Sierra Leone.

### The pharmaceutical industry in Sierra Leone before COVID-19: journey so far

With an estimated population of 8.3 million, Sierra Leone is a West African country along the Atlantic Ocean, and adjacent to Liberia and Guinea [7]. A recent data by West African Health Organization (WAHO) and United Nations Industrial Development Organization (UNIDO) reveal that Sierra Leone has no existing pharmaceutical manufacturing industry at present [8]. The data further reveal that there are no specific objectives and roadmaps currently in progress to establish pharmaceutical manufacturing companies in Sierra Leone [8]. Although a single manufacturing company had long existed in Sierra Leone, it had, however, ceased operations since the last two decades [8]. This dearth of pharmaceutical manufacturing capacity contributed significantly to the alarming rate of substandard and falsified medicines, and illegal importation of medicines into the country [8]. Hence, this is a signal to the central government to build, improve, and provide feasible access to locally manufactured products.

Before the advent of the COVID-19 pandemic in Africa [9], the Ebola outbreak had already affected many African countries such as Burkina Faso, Cape Verde, Ghana, Nigeria, Sierra Leone, etc., exposing the dysfunctional and weak state of the various pharmaceutical industries in these countries, including Sierra Leone [10]. During the Ebola epidemic, the Systems for Improving Access to Pharmaceuticals and Services (SIAPS) Program was established to rebuild the Sierra Leonean pharmaceutical industry, with operations beginning in September 2015 and under full awareness and permission of the central government of Sierra Leone [10]. It was a special project funded by the United States Agency International Development (USAID) to ensure access to essential medicines, proper healthcare facilities, and pharmaceutical products in the country. The SIAPS worked with the National Pharmaceutical Procurement Unit (NPPU) and the Directorate of Drugs and Medical Supplies (DDMS) to provide regulated policies, standard delivery, and better healthcare system during the Ebola period [10]. Notably, the Ebola outbreak brought to limelight the fragility and weakness of the Sierra Leonean pharmaceutical industry, which creates further uncertainties if the manufacturing capacity of the industry can withstand the effects of any potential disease outbreak such as the present COVID-19 pandemic.

### Impact of COVID-19 on medicines availability and drug importations in Sierra Leone

The response of the Sierra Leonean government to the COVID-19 pandemic was swift and decisive [11]. However, the impact of the pandemic on the local pharmaceutical industry was unprecedented as it resulted in severe drug shortages and medicine insecurity in the country due to the restrictions imposed on flights and cross-border importations by the government [11]. This incidence was also reported in other low- and middle-income countries [12]. Recall that there are currently no pharmaceutical manufacturing companies in Sierra Leone [8], compared to the Democratic Republic of Congo with 30 pharmaceutical manufacturers [13], Nigeria with more than 165 pharmaceutical manufacturers [14], and Zimbabwe with more than eight pharmaceutical manufacturers [15]. As a result, Sierra Leone is forced to depend solely on the importation of drugs and basic pharmaceutical commodities in a bid to meet its pharmaceutical needs and public health demands. Excessive importations like this, on the other hand, provide an opportunity for falsified and substandard medicines to enter into the legitimate medicine supply chain [11], as already witnessed in the Nigerian drug market [16]. The impact of the non-functional pharmaceutical industry in Sierra Leone was enormous during the Ebola outbreak and it was expected that the country would accumulate its resources to build and intensify local pharmaceutical manufacturing. The effects of the COVID-19 pandemic on drug availability and medicine security may point to the fact that concerted efforts were not taken or that building the local pharmaceutical industry was not taken as a public health priority. Given the possibility of a potential disease outbreak or any unprecedented incidence, this is yet another opportunity and a wake-up call for Sierra Leone to consolidate the lessons learnt from the Ebola outbreak and the COVID-19 pandemic, rethink the playbook, and devise strategies to build and revitalize the local pharmaceutical industry.

### What lessons can the Sierra Leonean pharmaceutical sector learn from COVID-19?

Due to the experiences associated with the COVID-19 pandemic, national governments throughout the African continent have demonstrated their political will to prioritize local pharmaceutical manufacturers over foreign manufacturers and suppliers, as long as they can sufficiently meet the needs and demands of the local health system. It is now widely recognized that establishing a functional local pharmaceutical industry can boost economic growth, provide employment, promote research, and improve access to medicines and pharmaceutical products in the case of potential disease outbreaks. As a result, a rise in foreign pharmaceutical companies looking to establish themselves in African countries to provide drugs and other essential medical commodities has taken place. This provides an incentive for Sierra Leone to key into this untapped potential and work towards achieving self-sufficiency in the manufacture of medicines and pharmaceutical products via the following recommendations:

1. Tax incentives must be provided to the country’s big importers to establish a local pharmaceutical industry.
2. Importers who have their pharmaceutical companies in countries like Nigeria and Ghana, must be encouraged to open sub-factory outlets in the country for local manufacturing.
3. A memorandum of understanding should be drawn up between the National Medicines Supply Agency in Sierra Leone and the proposed new pharmaceutical industry that they would be purchasing their products from as long as quality is assured.
4. Government land in the outskirts of the industrial cities should be provided for the proposed pharmaceutical industry.

## Conclusion

No health system can survive without a functional pharmaceutical industry, as access to essential medicines and pharmaceutical products are a cannot-do-without. Sierra Leone must, therefore, prioritize the establishment of a functional pharmaceutical industry based on lessons learnt from the recent COVID-19 pandemic. The recommendations provided in the context of this paper should be explored by the national government in order to safeguard the integrity and ensure self-sufficiency of the Sierra Leone pharmaceutical sector.

## Data Availability

Not applicable.

## Abbreviations

COVID-19: Coronavirus disease-2019
WHO: World Health Organization
WAHO: West African Health Organization
UNIDO: United Nations Industrial Development Organization
SIAPS: Systems for Improving Access to Pharmaceuticals and Services
USAID: United States Agency International Development
NPPU: National Pharmaceutical Procurement Unit
DDMS: Directorate of Drugs and Medical Supplies
